# Supplementary material for: Gender disparities in bladder cancer: A population-based study on life expectancy and health spending in Asia
Source: PLoS One. 2025 Jun 4;20(6):e0323803. doi: 10.1371/journal.pone.0323803 (PMC12136307; doi:10.1371/journal.pone.0323803)
Supplement: S1 Text — (DOCX) [file pone.0323803.s001.docx]

**S1 text.**

**The semiparametric survival extrapolation method used in this study involves three main steps:**

1. Creating a Reference Group: First, we created a reference group that was matched to patients with bladder cancer, based on age, sex, and the year of diagnosis. This group was generated by using the Monte Carlo method, which simulates lifespans, based on life tables from Taiwan's National Vital Statistics. The reference group provides a baseline for survival, showing how long people without bladder cancer are expected to live.
2. Comparing Survival Rates: Each month, we compared the survival rates of bladder cancer patients with those of the reference group. We calculated the ratio of these two survival rates and applied a logit transformation, which made the data follow a more linear pattern. This transformation helps in creating a smoother trend that was easier to model over time. We then used a restricted cubic spline model to estimate the survival rate of patients with bladder cancer for the next month, based on this trend.
3. Extending the Survival Curve: After estimating the survival rate for the next month, we repeated this process, extending the survival curve month by month. We continued until the survival rate was close to zero, indicating that nearly all patients had died. The area under this survival curve provided us the estimated life expectancy (LE) of patients with bladder cancer. The loss of LE was calculated by measuring the difference between the survival curves of the patients with bladder cancer and the reference group, thereby showing how much life expectancy was reduced because of the disease.
