# Supplementary material for: Gender disparities in bladder cancer: A population-based study on life expectancy and health spending in Asia
Source: PLoS One. 2025 Jun 4;20(6):e0323803. doi: 10.1371/journal.pone.0323803 (PMC12136307; doi:10.1371/journal.pone.0323803)
Supplement: S1 Table — (DOCX) [file pone.0323803.s002.docx]

**S1 Table. Validation of extrapolated life expectancy estimates in bladder cancer patients: comparison of 6-year follow-up extrapolations with 12-year Kaplan–Meier estimates.^a^**

| sex | age | Censoring rate  at end of 6th  year, % | Estimate using the extrapolation based on the first 6 years to the 12th year, months | Actual follow-up of 12 years by Kaplan-Meier estimate, months | Relative bias^b^, % |
| --- | --- | --- | --- | --- | --- |
| Male |  |  |  |  |  |
|  | 30-59 | 72.37 | 106.02(4.38) | 113.82(0.88) | -6.85 |
|  | 60-69 | 58.35 | 99.98(3.05) | 98.16(1.28) | 1.85 |
|  | 70-79 | 38.54 | 74.86(2.84) | 77.24(0.96) | -3.08 |
|  | 80-89 | 19.31 | 53.65(2.35) | 53.35(0.96) | 0.56 |
| Female |  |  |  |  |  |
|  | 30-59 | 68.33 | 106.03(4.41) | 108.32(1.68) | -2.14 |
|  | 60-69 | 57.68 | 93.76(6.63) | 95.77(1.83) | -2.1 |
|  | 70-79 | 37.32 | 74.86(3.44) | 73.64(1.64) | 1.66 |
|  | 80-89 | 15.99 | 45.34(4.08) | 44.54(1.71) | 1.8 |

^a^ Data from bladder cancer patients diagnosed between 2008 and 2013 were used for the extrapolation. Unless otherwise indicated, values are presented as mean (SEM).

^b^ Relative bias (%) = [(Extrapolated estimate – Kaplan–Meier estimate) / Kaplan–Meier estimate] × 100.
