# Supplementary material for: Gender disparities in bladder cancer: A population-based study on life expectancy and health spending in Asia
Source: PLoS One. 2025 Jun 4;20(6):e0323803. doi: 10.1371/journal.pone.0323803 (PMC12136307; doi:10.1371/journal.pone.0323803)
Supplement: S2 Table — (DOCX) [file pone.0323803.s003.docx]

**S2 Table. Multivariable cox proportional hazards regression analysis of overall survival in bladder cancer patients.**

| Variable |  | p-value | Hazard Ratio | 95% CI |
| --- | --- | --- | --- | --- |
| Sex | Female | - | reference | - |
|  | Male | <0.01 | 0.90 | 0.87-0.94 |
| Age | 30-59 | - | reference | - |
|  | 60-69 | <0.01 | 1.49 | 1.39-1.60 |
|  | 70-79 | <0.01 | 2.50 | 2.34-2.66 |
|  | 80-89 | <0.01 | 4.62 | 4.33-4.93 |
| Grade | Low | - | reference | - |
|  | High | <0.01 | 1.52 | 1.44-1.60 |
| Stage | 0-1 | - | reference | - |
|  | 2-4 | <0.01 | 2.72 | 2.62-2.84 |
