# Supplementary material for: Gender disparities in bladder cancer: A population-based study on life expectancy and health spending in Asia
Source: PLoS One. 2025 Jun 4;20(6):e0323803. doi: 10.1371/journal.pone.0323803 (PMC12136307; doi:10.1371/journal.pone.0323803)

**S1 Fig.**

**Comparison of life expectancy (LE) loss estimates across multiple cohorts.**

For female bladder cancer patients diagnosed at stage 1, their life expectancy after diagnosis is 11.74 years, which is 1.35 years shorter than that of male bladder cancer patients diagnosed at the same stage (whose life expectancy after diagnosis is 13.09 years). Life expectancy loss refers to the difference between the life expectancy in the study cohort and that of a reference population matched by age, sex, and calendar year, simulated using life tables. A comparison of life expectancy loss, or the difference in life expectancy loss (7.69−3.84 = 3.85), represents a difference-in-differences adjusted for potential confounding factors. Values are presented as mean ± SEM.


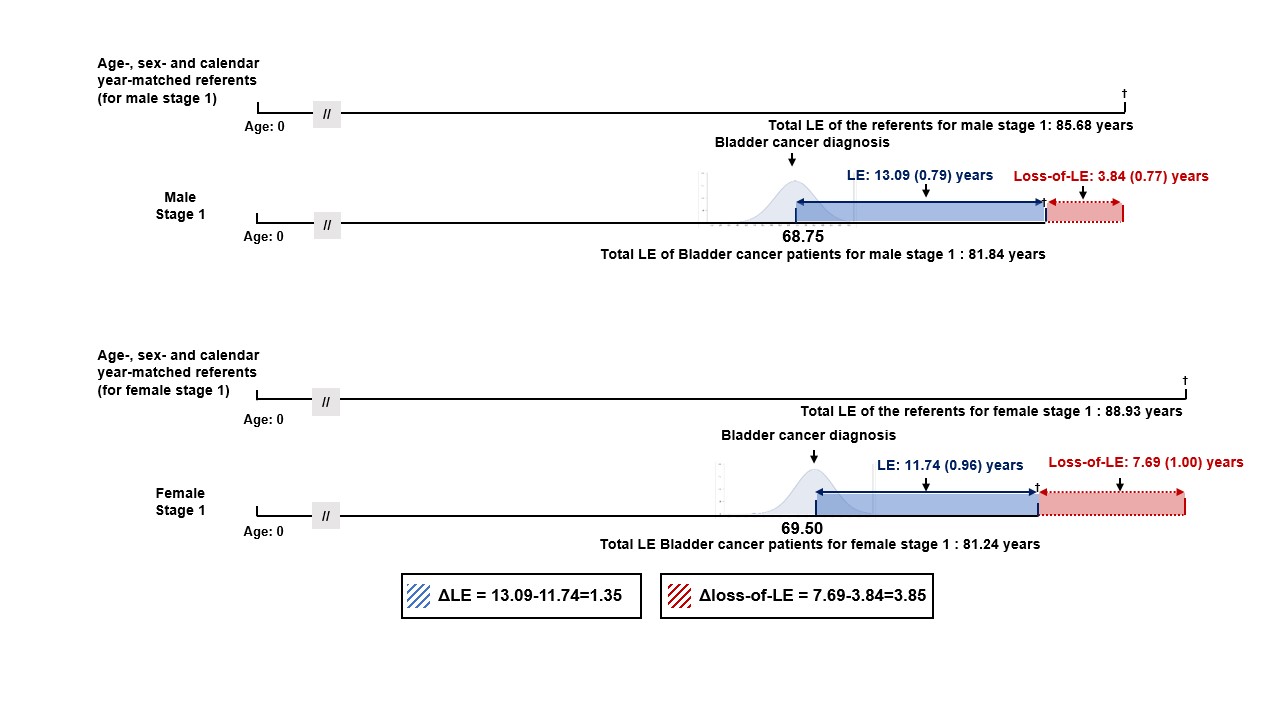

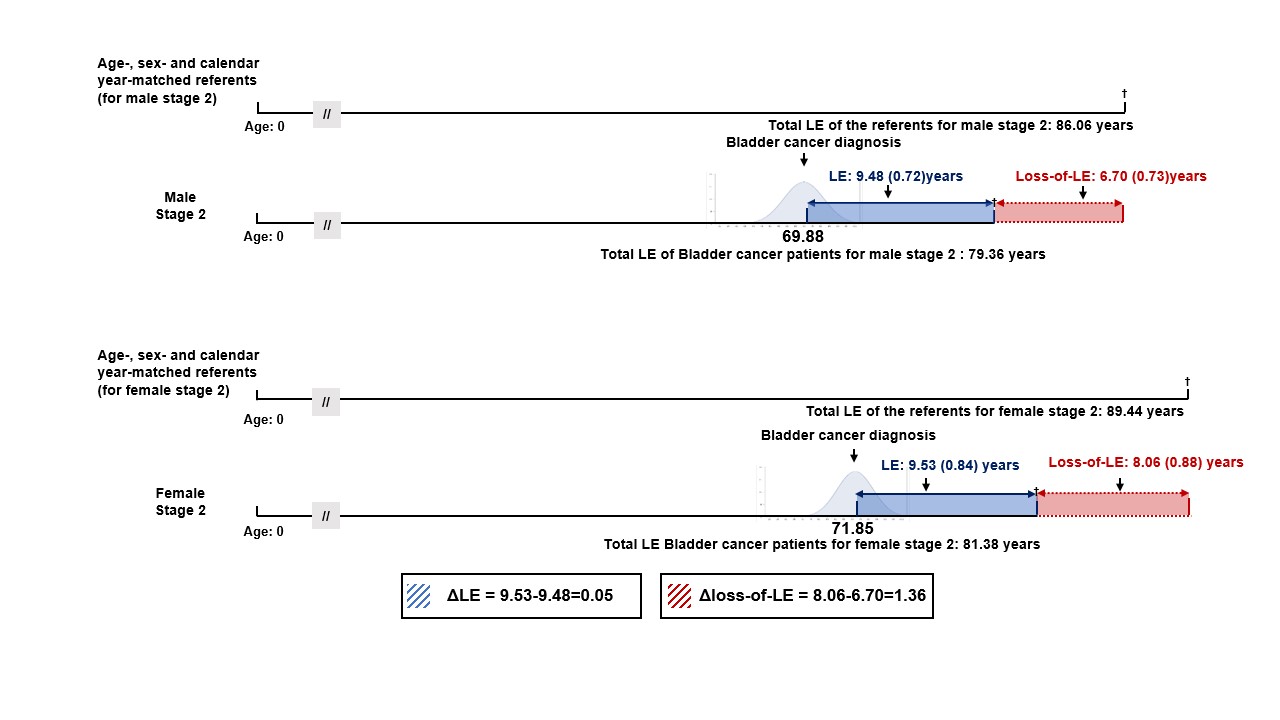

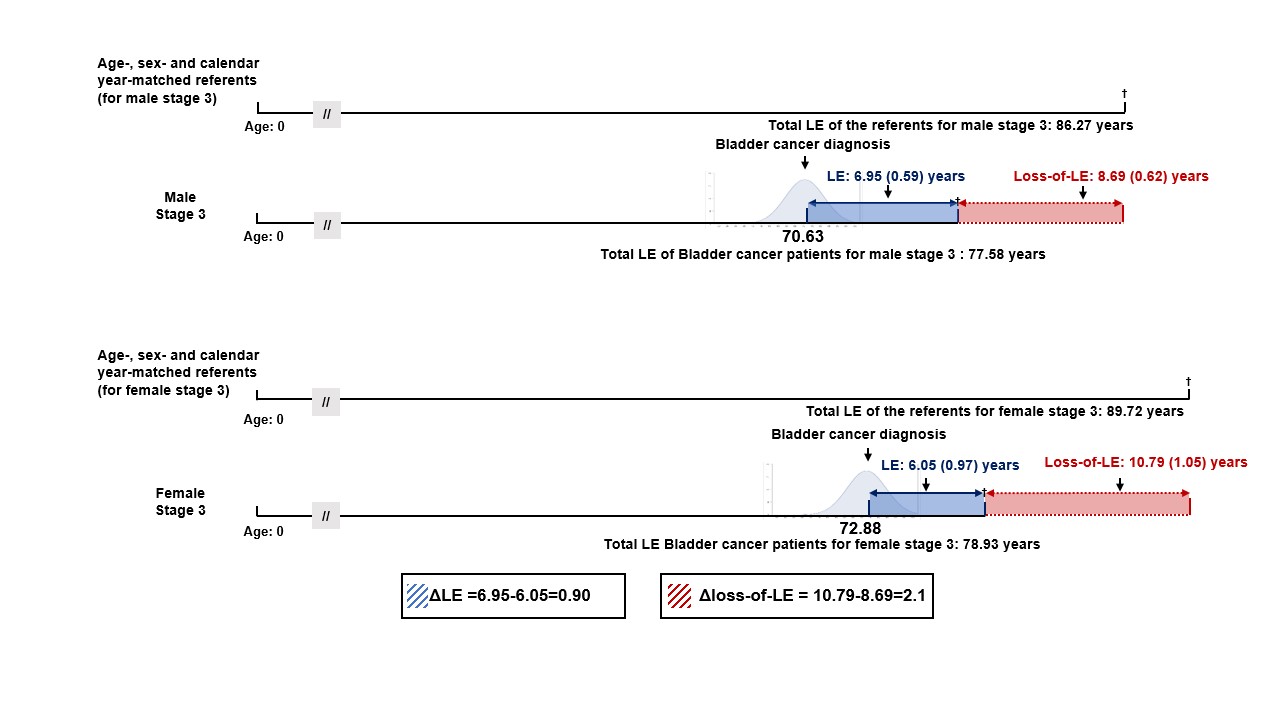

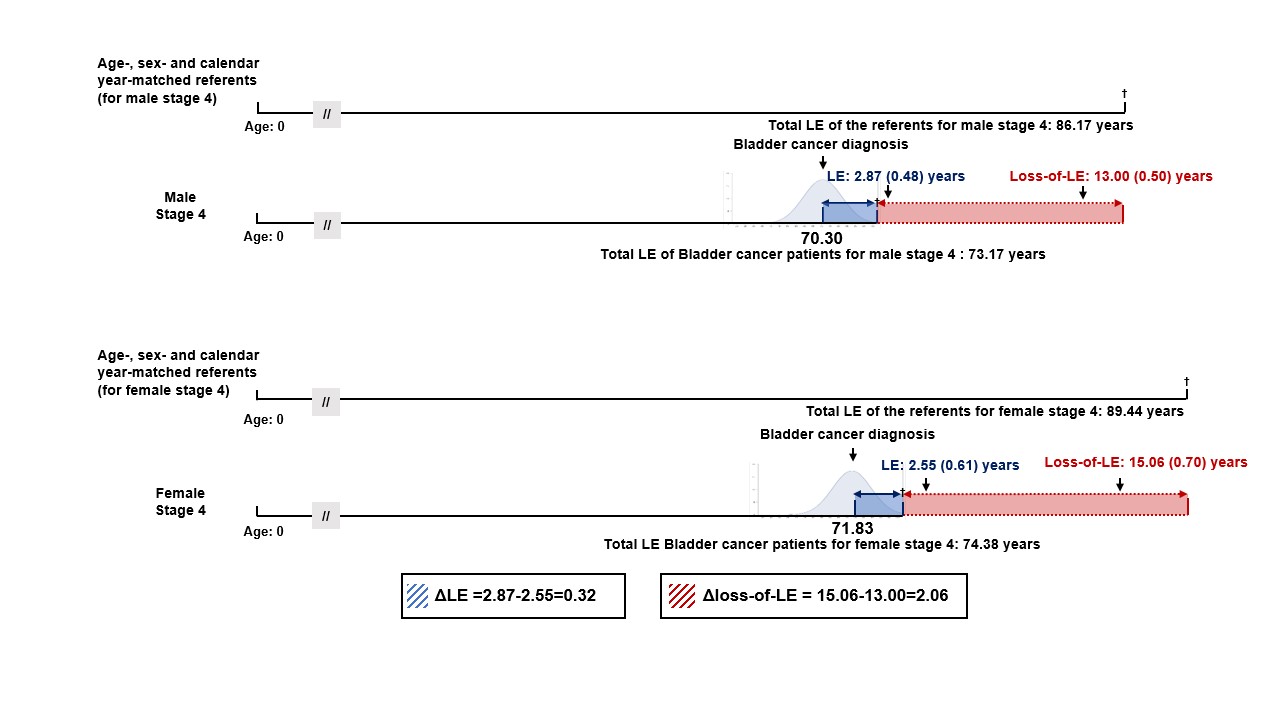

Supplement: S1 Fig — For female bladder cancer patients diagnosed at stage 1, their life expectancy after diagnosis is 11.74 years, which is 1.35 years shorter than that of male bladder cancer patients diagnosed at the same stage (whose life expectancy after diagnosis is 13.09 years). Life expectancy loss refers to the difference between the life expectancy in the study cohort and that of a reference population matched by age, sex, and calendar year, simulated using life tables. A comparison of life expectancy loss, or the difference in life expectancy loss (7.69 − 3.84 = 3.85), represents a difference-in-differences adjusted for potential confounding factors. Values are presented as mean ± SEM. (DOCX) [file pone.0323803.s004.docx]
