# Supplementary figures and images for: Gender disparities in bladder cancer: A population-based study on life expectancy and health spending in Asia
Source: PLoS One. 2025 Jun 4;20(6):e0323803. doi: 10.1371/journal.pone.0323803 (PMC12136307; doi:10.1371/journal.pone.0323803)

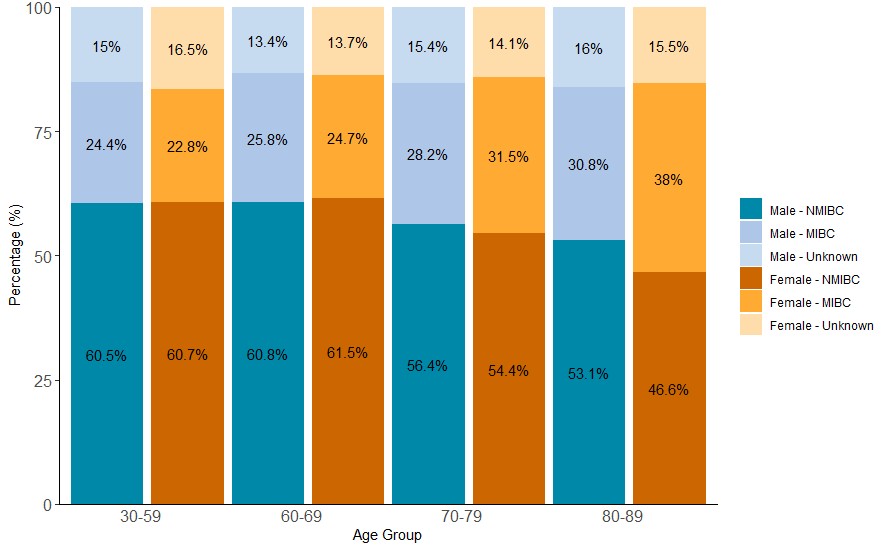

Supplement: S2 Fig — Both genders exhibited a similar trend, with the percentage of NMIBC decreasing and the percentage of MIBC increasing in older age groups. The proportion of cases classified as unknown remained consistent across all age groups and genders. In age groups below 70, females demonstrated a comparable or slightly higher percentage of NMIBC compared to males, whereas in age groups above 70, the percentage of NMIBC was significantly lower in females. Conversely, the percentage of MIBC showed an opposite pattern, with higher proportions observed in females in the older age groups. (JPG) [file pone.0323803.s005.jpg]

**S3 Fig.**

**Long term survival after bladder cancer diagnosis, stratified by sex and stage**


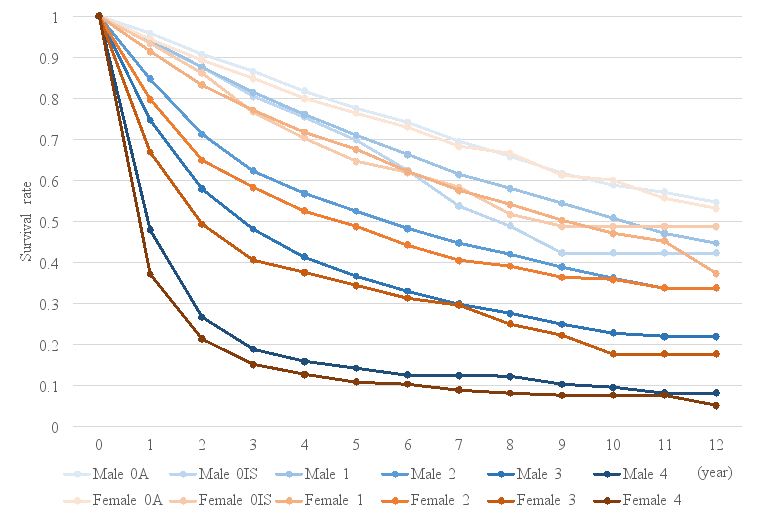

Supplement: S3 Fig — (DOCX) [file pone.0323803.s006.docx]
