# Supplementary material for: Gender disparities in bladder cancer: A population-based study on life expectancy and health spending in Asia
Source: PLoS One. 2025 Jun 4;20(6):e0323803. doi: 10.1371/journal.pone.0323803 (PMC12136307; doi:10.1371/journal.pone.0323803)
Supplement: Attachment — Submitted filename: Reviewer response.docx [file pone.0323803.s009.docx]

**Reviewer #1:**

Dear Author

Thank you for your revision. The items requested by me from the authors have been answered correctly and completely.

Answer:

We sincerely appreciate your time and effort in reviewing our manuscript. We are grateful for your positive feedback and for acknowledging that we have adequately addressed your previous comments. As per your assessment, no additional revisions were required. Nonetheless, we have carefully re-reviewed the entire manuscript to ensure clarity, accuracy, and completeness, and we have made further refinements based on feedback from the other reviewers to enhance the overall quality of our work.

Thank you again for your constructive review and support.

**Reviewer #2:**

The authors have properly addressed the comments, except for one. The death percentages in Table 2 appear to be incorrect. For instance, the percentage of deaths for low-grade females is listed as 159%. Other percentages in this table also seem incorrect, such as 1238 out of 6032 being reported as 74.98%. Please re-check the numbers in this table.

Answer:

We appreciate your careful review and for highlighting the discrepancies in Table 2. Upon re-examining our calculations, we discovered that errors arose from a misalignment in data extraction and an incorrect denominator for percentage calculations. We have thoroughly revised Table 2 (pages 12–13, revised Table 2) so that all reported values accurately reflect the proportion of deaths in each cohort. The corrected percentages now fall within expected ranges. Additionally, we reviewed all other tables and data sources to ensure consistency and accuracy throughout the manuscript.

Thank you again for your constructive feedback, which has significantly improved the precision of our study.

**Reviewer #3:**

1. While the study uses advanced statistical techniques, it does not report p-values for the individual components and comparisons within the semiparametric method. Calculating a single p-value for the entire extrapolation process is challenging due to the complexity and multi-step nature of the method. However, it is recommended to report p-values for individual components, such as the comparison of survival rates and covariates using Cox proportional hazards models, to provide a clearer understanding of the statistical significance of the findings.

Answer:

Thank you for your valuable feedback. In response, we have performed a multivariable Cox regression analysis including sex, age, pathological grade, and cancer stage (pages 8, lines 119–123). This analysis provides hazard ratios with 95% confidence intervals and p-values for each variable—for example, male sex is associated with a lower risk of death (HR 0.90, 95% CI 0.87–0.94, p < 0.01), while increasing age, high-grade tumors, and advanced-stage disease significantly elevate mortality risk (all p < 0.01). These results are detailed in the newly added Supplement Table S5 and summarized in the Results section (pages 16, lines 226–232, and new S2 Table). We believe this additional analysis enhances the statistical rigor and clarity of our findings.

2. The method assumes homogeneity in the patient data concerning the variables incorporated within the model. Variability in treatment, access to healthcare, and other patient-specific factors may not be comprehensively considered and ought to be recognized as a limitation.

Answer:

Thank you for this important comment. We acknowledge that our model assumes patient homogeneity for the included variables. In reality, variations in treatment, healthcare access, and other patient-specific factors may introduce residual confounding. We have revised the Limitations section to include the following statement (page 29, lines 458–460):

"Moreover, our model assumes patient homogeneity for the included variables. In reality, variations in treatment, healthcare access, and other factors may introduce residual confounding."

We believe this revision clearly recognizes the inherent heterogeneity in the patient population and enhances the transparency of our study.

3. Extending the survival curve month by month involves extrapolating beyond the observed data, introducing uncertainties. This approach assumes that the trends observed in the initial months will continue, which might not always be the case. Including sensitivity analyses or validating the model with external data could strengthen the findings.

Answer:

Thank you for your insightful comment. In response, we have inserted a sentence in the Methods section (page 8, lines 117–119; page 16, lines 221–226) describing a sensitivity analysis in which we compared life expectancy estimates extrapolated from the first 6 years of follow-up with 12-year Kaplan–Meier estimates (new Supplement S1 Table). The analysis revealed minimal relative bias, thereby supporting the robustness of our extrapolation approach despite inherent uncertainties. We hope this revision adequately addresses your concern.

4. The reference group created using the Monte Carlo method based on life tables may not fully capture all variables affecting the general population's survival, such as comorbidities or socio-economic factors. This could lead to biased comparisons and should be discussed as a potential limitation.

Answer:

Thank you for your valuable comment regarding our reference group. We recognize that using a Monte Carlo simulation based on life tables may not capture key factors—such as comorbidities and socioeconomic status—that affect the general population’s survival, potentially biasing comparisons. In response, we have revised the Limitations section (page 29, lines 457–458) to include the following statement:

"Additionally, the Monte Carlo-based reference group may not capture key factors (e.g., comorbidities, socioeconomic status), potentially biasing comparisons."

We believe this addition enhances the transparency of our study by clearly acknowledging this limitation.
